# Impact of nurse navigation and mobile app on brain tumor patients receiving oral anticancer therapy

**DOI:** 10.1038/s41746-025-02325-3

**Published:** 2026-01-15

**Authors:** Caroline Poisson, Adeline Duflot-Boukobza, Delphine Mathivon, Mohamed Khettab, Marie Ferrua, Aude Fourcade, Naïma Lezghed, Frédéric Dhermain, François Lemare, Vanessa Puglisi, May Abbas, Mario Di Palma, Florian Scotté, Etienne Minvielle, Olivier Mir, David Guyon, Sarah N. Dumont

**Affiliations:** 1 Department of Medical Oncology, Gustave Roussy, Université Paris-Saclay, Villejuif, France; 2 Division of Interdisciplinary Patient Care Pathways (DIOPP), Gustave Roussy, Université Paris-Saclay, Villejuif, France; 3 Medical Oncology, CHU de la Réunion, 97410, Université de La Réunion, Réunion, France; 4 Radiation Oncology Department, Gustave Roussy, Université Paris-Saclay, Villejuif, France; 5 Pharmacy Department,, Institut de Cancérologie de l’Ouest, Saint-Herblain, France

**Keywords:** Cancer, Oncology

## Abstract

Oral anticancer agents (OAAs) are commonly prescribed for patients with primary brain tumors, but adherence can be challenging due to cognitive impairment and discontinuous treatment schedules. This subgroup analysis of the randomized phase 3 CAPRI trial evaluated the impact of a nurse navigator-led intervention combined with a digital platform (web portal and mobile app) versus standard care in patients with primary brain tumors treated with OAAs. The primary endpoint was Relative Dose Intensity (RDI), with secondary endpoints including adherence, toxicity, healthcare utilization, and patient-reported experience. Fifty-one patients were included between October 2016 and May 2019, 63% of whom had glioblastoma. Twenty-six patients received the intervention. RDI was significantly higher in the intervention group compared to the control group (105% ± 12 vs. 97.6% ± 13, *p* = 0.04). The intervention also resulted in fewer emergency room visits, reduced hospitalizations, greater use of supportive care services, and improved patient-reported experience (all *p* < 0.05). Remote monitoring allowed early corticosteroid adjustments in cases suggestive of intracranial hypertension, helping to prevent hospitalizations. No significant differences were observed in treatment-related toxicity. These findings suggest that a nurse navigator-led digital intervention can improve care continuity and outcomes in this population and merit further investigation.

## Introduction

Glioblastoma Multiforme (GBM) is the most frequent primary malignant brain tumor in adults. It predominantly affects male patients, with the highest incidence occurring around the age of 65^1^. The overall prognosis remains poor, and long-term survival is rare. For patients with good performance status (Karnofsky performance status ≥60), the median overall survival (OS) is 14.6 months with concomitant radio-chemotherapy^2^. Between 8% and 67% of individuals diagnosed with glioma experience alterations in behavior and personality^3,4^. Patients with grade 3 astrocytomas and GBM present symptoms such as increased intracranial pressure, seizures, focal neurologic deficits, and changes in personality and mood. Cognitive impairment can also result from treatment-related adverse events, including radiation encephalopathy or chemotherapy-induced neurotoxicity. Moreover, the morbidity associated with the progressive deterioration of neurological function and quality of life (QoL) can have a devastating impact on both patients and their families. Supporting family caregivers is essential to help them continue their caregiving roles and maintain the best possible QoL for patients. Efforts to enhance family caregiver well-being are crucial and the use of digital tools to monitor the situation at home can be a precious asset.

Oral anticancer agents (OAAs) are frequently used in neuro-oncology. Unlike patients receiving intravenous chemotherapy, who typically visit the hospital frequently for treatment every 1–3 weeks depending on the chemotherapy regimen, patients receiving OAAs are generally monitored less frequently during scheduled consultations. Nonadherence is a significant issue in neuro-oncology as the use of oral cytotoxic agents increases. Several studies have highlighted challenges in maintaining adherence due to cognitive impairment, complex regimens, and treatment-related side effects in patients with brain tumors^5–7^.

In oncology, studies have suggested that remote patient monitoring could help managing chemotherapy-related adverse events^8^ and improve adherence to OAAs^9^. These studies typically involved nurse-led monitoring through phone calls and emails. However, the impact of digital remote monitoring systems in this setting was not well evaluated. From 2016 to 2019, Gustave Roussy conducted a phase 3 study (CAPRI, NCT02828462)^10^, aimed to assess the impact of an intervention combining a nurse navigator-led follow-up and a mobile application for patients receiving OAA on top of standard of care. The study’s primary outcome was Relative Dose Intensity (RDI). Secondary outcomes included the incidence of grade ≥3 toxicities, patient-reported experience, hospitalization frequency and duration, treatment response rate, progression-free survival, overall survival, and QoL. Among 559 evaluable participants, the RDI was significantly higher in the intervention group compared to the control group (93.4% vs. 89.4%, *p* = 0.04). The intervention also improved the patient experience (Patient Assessment of Chronic Illness Care score^11^, 2.94 versus 2.67, *p* = 0.01), decreased the number of days of hospitalization (2.82 versus 4.44 days, *p* = 0.02), and decreased the prevalence of grade ≥3 toxicities (27.6% versus 36.9%, *p* = 0.02). These findings showed that patient-centered care through remote monitoring of symptoms and treatment may improve patient outcomes and experience.

We herein present a subgroup analysis of outcomes in patients with primary brain tumors, who represent one of the most vulnerable oncology populations due to cognitive impairment, with a critical role played by their proxies.

## Results

### Patient characteristics

The subgroup of patients with primary brain tumors in the randomized study comprised 51 participants. In terms of demographic characteristics, both groups were broadly similar with respect to gender and oncological treatment, with temozolomide being the most commonly used agent. The CAPRI group exhibited greater tumor diversity, with a lower proportion of GBM compared to the other group. This could partly be explained by a younger average and median age in the CAPRI group. However, GBM remained the most common histology in both groups, consistent with the epidemiology of neuro-oncology populations. The proportion of family caregivers present in both groups was comparable, and the follow-up duration was also similar. Performance status (PS) was not fully balanced, as a higher proportion of patients in the CAPRI arm had PS = 0 (Table 1).

**Table 1:** Patients characteristics

	CAPRI (*n* = 26)	Control (*n* = 25)
**Demographic data**		
Female Median age [min–max]	13 (50) 51.5 [24–80]	14 (53.8) 58.5 [37–80]
**Performance status at enrollment**		
0 1 2	11 (42.3) 9 (34.6) 6 (23.1)	6 (24) 16 (64) 3 (12)
**Oncology data**		
Tumor type		
Glioblastoma Grade 3 Astrocytoma Others^a^	13 (50) 5 (19.2) 8 (30.8)	19 (76) 1 (4) 5 (20)
OAA		
Temozolomide Others^b^	23 (88.5) 3 (11.5)	24 (96) 1 (4)
Other treatment		
Corticosteroid therapy Anti-epileptic	13 (50) 17 (65.4)	10 (40) 17 (68)
**Presence of family caregivers**	23 (88.5)	22 (88)
**Duration of follow-up (days)**		
Min–max Mean Median	30–211 151 181	29–195 151 181

Data are expressed as *n* (%), median [min–max].

^a^Grade 2 Astrocytoma; Grade 2 and 3 Oligodendroglioma.

^b^Procarbazine,Lomustine, Everolimus.

### Treatment exposure and relative dose intensity

The RDI was significantly higher in the CAPRI group, with a mean of 105% ± 12 compared to 97.6% ± 13 in the control group (*p* = 0.04). This difference remained statistically significant when RDI was adjusted for treatment adherence (assessed using a dedicated questionnaire^12^) with a mean of 98.5% ± 14.5 in the CAPRI arm vs. 88% ± 16.9 in the control arm (*p* = 0.03) (Table 2).

**Table 2:** Treatment disposition and outcomes

Variable	CAPRI	CONTROL	Total	Statistical significance
**RDI (until study discontinuation)**^**a**^				
No. of patients	26	25	51	***t*** = **4.54**
Mean (S.d.)	1.0504 (0.1175)	0.9763 (0.1309)	1.0141 (0.1286)	***p*** = **0.04**
95% CI	[1.0030; 1.0979]	[0.9223; 1.0304]	[0.9779; 1.0503]	
Min–Max	[0.71; 1.24]	[0.56; 1.18]	[0.56; 1.24]	
Median	1.00	1.00	1.00	
Q1–Q3	[1.00; 1.16]	[1.00; 1.00]	[1.00; 1.09]	
**RDI (until study discontinuation) adjusted for global adherence**				
No. of patients	24	22	46	***t*** = **5.11**
Mean (S.d.)	0.9854 (0.1446)	0.8809 (0.1687)	0.9355 (0.1636)	***p*** = **0.03**
95% CI	[0.9243; 1.0465]	[0.8061; 0.9558]	[0.8869; 0.9840]	
Min–Max	[0.7; 1.2]	[0.5; 1.1]	[0.5; 1.2]	
Median^b^	1.0	0.9	1.0	
Q1–Q3	[0.9; 1.1]	[0.8; 1.0]	[0.8; 1.1]	
**Adherence,** ***n*** **(%)**^**c**^				
No. Of patients	24	22	46	*X* = 0.58
High	4 (16.7%)	2 (9.1%)	6 (13.0%)	*p* = 0.75
Medium	19 (79.2%)	19 (86.4%)	38 (82.6%)	
Low	1 (4.2%)	1 (4.5%)	2 (4.3%)	
**Response rate at 6 months**				
Missing	2 (7.7%)	0	2 (3.9%)	*X* = 2.19
No. of patients	24	25	49	*p* = 0.07
Stable disease	20 (83.3%)	**15 (60.0%)**	**35 (71.4%)**	
Progressive disease	4 (16.7%)	10 (40.0%)	14 (28.6%)	

^a^RDI was defined as the ratio of the dose actually delivered over time to the planned dose intensity. Values above 100% can occur due to early initiation of cycles, absence of dose reductions, or rounding of oral formulations.

^b^Regarding RDI reporting : The median is identical across groups, reflecting the small sample size and the discrete distribution of values, but the mean allows detection of subtle differences in overall dose delivery and variability, which were statistically significant.

^c^Adherence was categorized as high, medium, or low based on the validated Morisky scale and/or Medication Event Monitoring System (MEMS) thresholds.

The values in bold indicate statistically significant results (*p* < 0.05).

### Toxicities and clinical outcomes

However, there was no significant difference in grade 3 and 4 toxicity rates or adherence between the two groups. Additionally, the CAPRI group experienced fewer emergency consultations (2 vs 14, *p* = 0.02) and a lower number of patients requiring emergency consultations prior to hospitalization (1 vs 6, *p* = 0.0496), suggesting improved clinical outcomes in this cohort (Table 3). This remote follow-up of patients with brain tumors enabled an early adjustment of the dose of corticosteroids in the case of symptoms suggestive of intracranial hypertension, and to avoid a hospitalization.

**Table 3:** Toxicities and hospitalizations per treatment arm

Variable	CAPRI	Control	Total	Statistical significance
**Grade** ≥ **3** **toxicities**				
No. of patients	26	25	51	*X* = 0.4
No	20 (76.9%)	21 (84.0%)	41 (80.4%)	*p* = 0.52
Yes	6 (23.1%)	4 (16.0%)	10 (19.6%)	
**Grade 1 and 2 toxicities**				
No. of patients	26	25	51	*X* = 2.19
No	3 (11.5%)	7 (28.0%)	10 (19.6%)	*p* = 0.14
Yes	23 (88.5%)	18 (72.0%)	41 (80.4%)	
**Number of emergency room visits**				
No. of patients	26	25	51	*T* = 6.07
No	24 (92.3%)	11 (44.0%)	35 (68.6%)	***p*** = **0.02**
Yes	2 (7.7%)	14 (56.0%)	16 (31.4%)	
**No. of hospitalizations after ER visits**				
No. of patients	26	25	51	*T* = 0.0398
No	25 (96.2%)	19 (76.0%)	44 (86.3%)	***p*** = **0.0496**
Yes	1 (3.8%)	6 (24.0%)	7 (13.7%)	
**No. of hospitalizations**				
No. of patients	26	25	51	*T* = 3.07
No	22 (84.6%)	16 (64.0%)	38 (74.5%)	*p* = 0.09
Yes	4 (15.4%)	9 (36.0%)	13 (25.5%)	

Data are expressed as *n* (%).

The values in bold indicate statistically significant results (*p* < 0.05).

### Use of supportive care services

Whereas 7 patients (28%) in the control arm had at least one ambulatory visit with supportive care teams during the study, 15 (57.7%) did so in the CAPRI arm (*p* = 0.03). Specifically, a significant increase was seen in visits to social workers (11 vs 4, *p* = 0.01) (Table 4).

**Table 4:** Patient receiving supportive care and type of supportive care

	CAPRI	CONTROL	Total	Statistical significance
No. of patients	26	25	51	
Receiving supportive care	15 (57.7)	7 (28)	22 (43.1)	***p*** = **0.03**
Pain	0	0	0	*p* = 1.00
Palliative care	1 (3.8)	0	1 (2)	*p* = 0.65
Nutrition	1 (3.8)	0	1 (2)	*p* = 0.65
Psychological support	15 (57.7)	9 (36)	24 (47.1)	*p* = 0.12
Social workers	11 (42.3)	4 (16)	25 (49)	***p*** = **0.01**

Data are expressed as *n* (%).

The values in bold indicate statistically significant results (*p* < 0.05).

### Patient-reported experience of care

Finally, the CAPRI intervention improved the patient experience of care. The mean (s.d.) global PACIC score was 3.4 (0.54) in the CAPRI arm and 2.84 (0.92) in the control arm (*p* = 0.0002) (Table 5). Eighty-three percent of patients reported being satisfied with the CAPRI intervention, feeling “listened to” and “secured”. Many patients also expressed that they were better able to manage side effects, highlighting the positive impact of the intervention on both care access and patient satisfaction.

**Table 5:** Patient experience (PACIC score) per treatment arm

Dimension	CAPRI	CONTROL	Total	Statistical significance
Global score				
Missing, *n* (%)	13 (50.00)	15 (60.00)	28 (54.90)	*t* = 20.76
No. of patients	13	10	23	*p* = 0.0002
Mean (S.d.)	3.40 (0.54)	2.12 (0.80)	2.84 (0.92)	
95% CI	[3.07; 3.73]	[1.54; 2.70]	[2.45; 3.24]	
Min–Max	[2.50; 4.30]	[1.20; 3.60]	[1.20; 4.30]	
Median	3.50	1.90	3.10	
Q1–Q3	[3.10; 3.70]	[1.50; 2.35]	[2.05; 3.60]	

Data are expressed as *n* (%).

### Nurse navigator activities and remote monitoring engagement

**Fig. 1:**
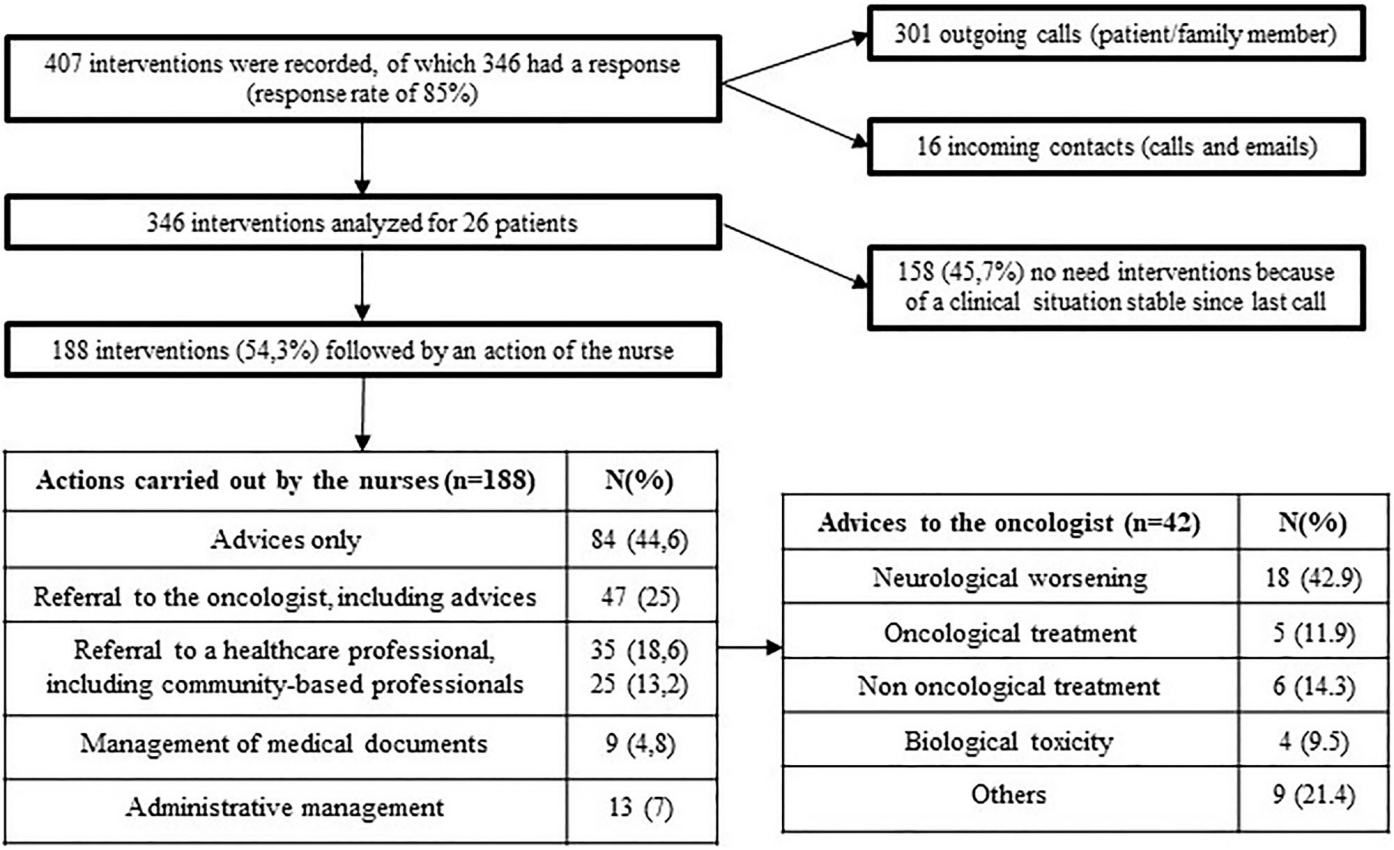
Descriptive results of the Nurses’activity.

A total of 407 interventions were carried out by NN for 26 patients (i.e., an average of 15.7 interventions per patient) during the follow-up period (with a maximum follow-up period of 6 months). The rate of response to these calls was 85%, indicating adherence to the device. Of the interventions, 54.3% were followed by an action from the nurses, while 45.7% involved no specific action, meaning there was either no new toxicity or no worsening of pre-existing toxicities during follow-up. A referral to the referring oncologist was made in 25% of follow-ups where action by the nurses was required (Fig. 1).

## Discussion

This subgroup analysis explored the potential impact of the CAPRI intervention—a program combining nurse navigator-led follow-up with a mobile health application—on patients with primary brain tumors receiving oral anticancer therapies, in comparison to standard care alone. The trial showed a statistically significant increase in RDI in the intervention group (105% (±0.12) vs. 97.6% (±0.13), *p* = 0.04), though the clinical relevance of this difference warrants careful interpretation. In routine clinical practice, temozolomide dosing is subject to inherent variability due to capsule rounding, which often results in deviations from the intended dose. Within this context, the observed 7.4% difference between groups may fall within the range accepted in everyday clinical care. Nonetheless, after adjustment for adherence, RDI remained significantly higher in the intervention arm (98.5% ± 0.14 vs. 88.1% ± 0.17, *p* = 0.03), underscoring that the CAPRI program may contribute to improved treatment continuity.

The outgoing call activity from the NN was the main activity, and the discontinuous nature of oral treatment (e.g., 5 days on a 28-day cycle) leads to a considerable risk of missed doses. Through these calls, adherence was significantly higher in the CAPRI group. Interestingly, these findings were not corroborated by the Morisky adherence scale^12^, raising questions about the appropriateness of such tools in this patient population with discontinuous medications. These results should be interpreted cautiously given the small sample size, the baseline imbalances in glioblastoma prevalence and performance status between groups (PS and glioblastoma prevalence favoring the CAPRI arm).

Despite the significantly positive RDI, we did not observe an impact on tumor response. However, the proportion of patients with no progression favored the CAPRI group at the significance threshold (83.3% non-progression at 6 months in the CAPRI group vs. 60%, *p* = 0.07). It should be noted that tumor response in the main CAPRI trial was assessed using RECIST 1.1 across all tumor types. However, for the subgroup of patients with brain tumors, RECIST is not the most appropriate tool; RANO or RANO 2.0 criteria would provide a more suitable framework, although no central RANO review was conducted.

Importantly, the CAPRI study echoes broader findings from other oncology domains, notably the landmark randomized trial by Temel et al.^13^ in advanced non-small cell lung cancer, which demonstrated that early integration of palliative care not only improved quality of life and mood but also significantly prolonged OS. This unexpected survival benefit, independent of anticancer treatment intensification, suggests that structured supportive care may influence outcomes beyond symptom control alone. In this context, the improved RDI observed in the CAPRI study may represent a complementary mechanism through which supportive interventions exert clinical benefit - by promoting treatment adherence and continuity. Moreover, our findings are consistent with and build upon prior work by Ethan Basch and colleagues^14–16^, who demonstrated that remote monitoring of patient-reported symptoms during cancer treatment can significantly improve clinical outcomes, including quality of life, reduced emergency visits, and even overall survival. While Basch’s work primarily focused on intravenous therapies and broad oncology populations, our study extends this evidence to a neuro-oncology subgroup receiving oral anticancer agents, incorporating not only symptom monitoring but also personalized support via nurse navigators and a mobile app. This integrated approach highlights the added value of digital health interventions in complex cancer care settings, particularly where cognitive and adherence challenges are common.

The first contact between the patient and the CAPRI NNs occurred during an in-person consultation, which helped establish a “human” connection while also introducing the system and evaluating the patient. At the beginning of the follow-up, a vulnerability screening was performed by the NNs. Autonomous follow-up by NNs accounted for 75% of contacts. This required predefined clinical protocols and escalation pathways, developed prior to the study in collaboration with oncologists. The importance of these frameworks is heightened in the current context of limited medical staffing. Weekly meetings were scheduled with the neuro-oncologist to discuss patients. This study supports the impact of nurse-led follow-up, which could be carried out by an Advanced Practice Nurse (APN) through teleconsultations and regular follow-up visits. APNs are increasingly recognized across Europe as essential healthcare providers, helping to address workforce shortages and improve care delivery. As defined by the International Council of Nurses in 2020^17^, APNs are nurses with advanced clinical training and enhanced decision-making skills tailored to their specific practice contexts^18^. As the World Health Organization projects a global shortfall of 12.9 million healthcare professionals by 2035, APNs are positioned to play a critical role in mitigating provider shortages, particularly in underserved areas. Regarding oncologist consultations, a significant portion of requests were related to neurological deterioration. In the control group, the main reason for emergency department visits and hospitalizations was also neurological worsening. It is reasonable to assume that there is a cause-and-effect relationship between these two factors. Indeed, early detection of complications leading to an oncologist consultation (without waiting for a regular consultation) helps avoid emergency visits and hospitalizations, reducing costs without increasing the frequency of medical consultations.

This study underscores the potential value of supportive interventions in neuro-oncology. It is notable that the CAPRI group had significantly greater access to supportive care, particularly in terms of access to social services. Given the poor prognosis of GBM, the most prevalent pathology in both groups, it appears essential to provide supportive services as early as possible. However, outcomes related to family caregivers were not evaluated. Given their pivotal role in ensuring treatment adherence and providing daily support, future research should incorporate caregiver-centered endpoints, such as caregiver satisfaction, burden, and overall well-being. In particular, assessing both emotional and physical signs of caregiver burnout would offer valuable insights. It is reasonable to assume that early symptom management and the timely initiation of home care services have a significant impact on caregivers. As with patients, the availability of a dedicated point of contact—such as a nurse—can be a major source of reassurance for family caregivers.

Both the CAPRI study’s neuro-oncology subgroup analysis and the recent work by Minvielle et al.^19^ highlight the value of combining nurse navigator-led interventions with digital tools to enhance cancer care. While the CAPRI study demonstrated that this hybrid approach significantly improved treatment adherence (as reflected by higher RDI), reduced emergency visits and hospitalizations, and enhanced patient-reported outcomes in patients with brain tumors, Minvielle et al. used structural equation modeling to show that the quality of interactions with nurse navigators was directly associated with improved patient satisfaction and reduced acute care use. Together, these studies provide complementary evidence that digital remote monitoring, when integrated with personalized human support, can significantly optimize treatment delivery and continuity of care in oncology. Moreover, the CAPRI study also demonstrated a significant medico-economic impact. The intervention led to a reduction in emergency department visits and hospitalizations. Importantly, this decrease in acute care utilization was not offset by an increased use of community-based healthcare services, such as general practice or outpatient consultations^20^.

However, another limitation is that the CAPRI trial excluded patients without access to phone or internet, potentially limiting generalizability. This raises important concerns about digital and health equity. Future efforts should ensure that digital health models are accessible to patients with limited digital literacy or resources.

Although the observed improvement in RDI lends support to the role of the CAPRI intervention in sustaining treatment continuity, its broader clinical impact is more convincingly reflected in secondary outcomes. The reduction in emergency department visits and hospitalizations, coupled with measurable gains in patient‑reported outcomes, and better access to supportive care, provides stronger evidence of tangible benefit. Taken together, these findings underscore the added value of integrating nurse navigator-led digital support into neuro‑oncology care, where continuity, early complication management, and patient experience are critical dimensions of quality. While promising, these results are exploratory and hypothesis-generating, and should be confirmed in larger, dedicated trials with caregiver and equity-focused endpoints. Looking ahead, the integration of APNs may represent a logical evolution of this model. Their expanded scope of practice could allow for greater autonomy in managing symptom-related treatments and reduce the reliance on oncologists for routine interventions—offering a promising path forward in the context of increasing healthcare system pressures.

## Methods

### Study design and setting

This is a subgroup analysis of patients with primary brain tumors from the broader cohort of patients included in the original CAPRI study, which involved all patients with solid tumors. The CAPRI study was a single-center (Gustave Roussy Comprehensive Cancer Center) randomized phase 3 trial designed to evaluate the impact of the ‘CAPRI’ navigation program, which involved two Nurse Navigators (NN) and a mobile application, compared to standard care (SoC) in the routine delivery of oral anticancer agents (OAA). The study protocol has been previously published elsewhere^10,21^. The main inclusion criteria were: age ≥18 years, PS score ≤2, and initiation of oral anticancer therapy, regardless of treatment line. After providing informed consent, patients were randomized into either the CAPRI intervention group or the control group. Randomization was stratified by pathology and PS score to ensure balanced distribution. At the end of the 6‑month follow‑up, patients in the control group were allowed to request CAPRI follow‑up.

Although eligibility for the CAPRI trial required patients to have advanced or metastatic cancer, this subgroup analysis focused exclusively on patients with primary brain tumors (e.g., glioblastoma, astrocytoma, oligodendroglioma). While these tumors do not metastasize in the conventional sense, their locally aggressive behavior and the need for systemic oral therapy justified their inclusion within the broader trial population.

### Intervention

**Fig. 2:**
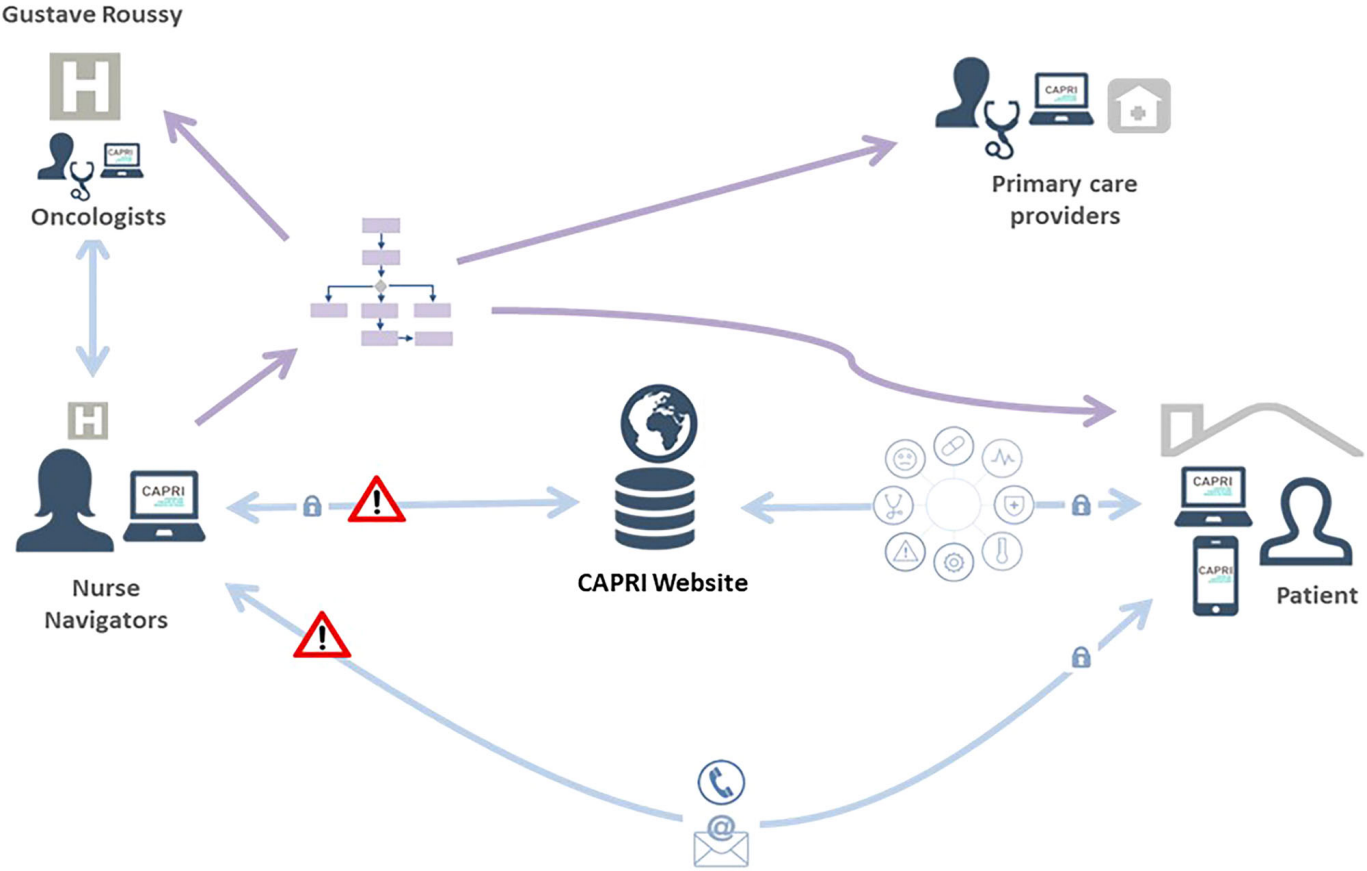
Navigation program « CAPRI ».

During the study, patients from the control group received usual care (consultations with the treating oncologist at Gustave Roussy, mainly for prescription renewals and dose modifications), while participants from the intervention group benefited from the CAPRI program on top of similar usual care. The digital remote monitoring tools used in the CAPRI trial included a web portal and a mobile application, both accessible to patients, nurse navigators, and caregivers. The NN called the patient every week for the first month and then decreased the follow-up frequency according to the protocol. They evaluated the symptoms, the need of supportive care and graded the toxicities. The patient could alert the NN either through the application or with a dedicated phone line (from Monday to Friday, from 9 AM to 5 PM). To treat the alerts, grading and orientation neuro-oncology-tailored algorithms have been developed. Family practitioner and private nurses were involved from the beginning of the follow-up and access to the application was created for them. Patients could record tracking data, contact nurse navigators via secured messaging, access to medical information or store documents. The duration of the intervention was 6 months (Fig. 2).

### Endpoints

The primary endpoint was the Relative Dose Intensity (RDI) at 6 months defined as the ratio of the actual delivered dose to the planned theoretical dose over a specified treatment period. Values exceeding 100% may arise due to the calculation method, for instance when treatment cycles start slightly earlier than scheduled, when no dose reductions or interruptions occur in patients with high adherence, or when oral formulations necessitate dose rounding that results in a slightly higher administered dose.

Secondary endpoints were: patient adherence to OAA (measured with a dedicated questionnaire and/or the Medication Event Monitoring System (MEMs)), patient experience (PACIC score), tumor response (evaluated by investigators, using RECIST 1.1), grade ≥3 toxicities (graded according to the NCI-CTCAE v4.03 classification).

### Statistical analysis

Descriptive statistics were used, including median (range) and mean (±SD) values, interquartile ranges and 95% confidence intervals; comparisons were made using Chi-square and Student’s *t* test when appropriate.

### Ethics approval and consent to participate

The study was conducted in agreement with applicable laws and regulations and the Declaration of Helsinki, and was approved by an ethics committee (CPP Paris-Ile-de-France IV no. 2016/20SC, US Department of Health and Human Services approved IRB no. 00003835). Written informed consent was obtained from all participants prior to inclusion in the study.

## Data Availability

The datasets generated and/or analyzed during the current study are available from the corresponding author upon reasonable request.
